# Substituted Caffeic and Ferulic Acid Phenethyl Esters: Synthesis, Leukotrienes Biosynthesis Inhibition, and Cytotoxic Activity

**DOI:** 10.3390/molecules22071124

**Published:** 2017-07-06

**Authors:** Pier Morin, Patrick-Denis St-Coeur, Jérémie A. Doiron, Marc Cormier, Julie J. Poitras, Marc E. Surette, Mohamed Touaibia

**Affiliations:** Department of Chemistry and Biochemistry, Université de Moncton, Moncton, NB E1A 3E9, Canada; pier.morin@umoncton.ca (P.M.J.); eps7653@umoncton.ca (P.-D.S.-C.); jeremie.doiron@umoncton.ca (J.A.D.); marc.cormier@umoncton.ca (M.C.); ejp6815@umoncton.ca (J.J.P.); marc.surette@umoncton.ca (M.E.S.)

**Keywords:** glioblastoma multiforme, 5-lipoxygenase, temozolomide, polyphenols

## Abstract

Glioblastoma multiforme (GBM) is an aggressive brain tumor that correlates with short patient survival and for which therapeutic options are limited. Polyphenolic compounds, including caffeic acid phenethyl ester (CAPE, **1a**), have been investigated for their anticancer properties in several types of cancer. To further explore these properties in brain cancer cells, a series of caffeic and ferulic acid esters bearing additional oxygens moieties (OH or OCH_3_) were designed and synthesized. (CAPE, **1a**), but not ferulic acid phenethyl ester (FAPE, **1b**), displayed substantial cytotoxicity against two glioma cell lines. Some but not all selected compounds derived from both (CAPE, **1a**) and (FAPE, **1b**) also displayed cytotoxicity. All CAPE-derived compounds were able to significantly inhibit 5-lipoxygenase (5-LO), however FAPE-derived compounds were largely ineffective 5-LO inhibitors. Molecular docking revealed new hydrogen bonds and π-π interactions between the enzyme and some of the investigated compounds. Overall, this work highlights the relevance of exploring polyphenolic compounds in cancer models and provides additional leads in the development of novel therapeutic strategies in gliomas.

## 1. Introduction

Glioblastoma multiforme (GBM) is the most frequent brain tumor diagnosed in adults and current standard of care, consisting of surgery, radiotherapy, and chemotherapy, translates to overall patient survival between 7 and 15 months [1]. Therapeutic options are desperately needed for a tumor that is associated with substantial genetic heterogeneity and in which drug resistance is often encountered [2,3]. Targeted therapies undertaken so far have notably aimed at frequently deregulated molecular players in GBM including EGFR and mTOR [4]. Gliomas have also likely developed strategies to inhibit adaptive immune responses ultimately contributing to tumor cell survival and those are also under investigation [5].

**Figure 1:**
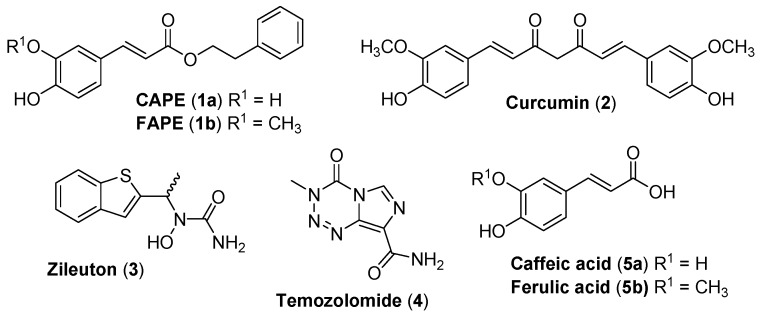
Structures of caffeic acid phenethyl ester (CAPE) (**1a**), ferulic acid phenethyl ester (FAPE) (**1b**), Curcumin (**2**), Zileuton (**3**), Temozolomide (**4**), Caffeic acid (**5a**), and ferulic acid (**5b**).

Several studies have assessed the therapeutic properties of natural or synthetic polyphenolic compounds in various cancers [6,7]. In addition, the chemo-sensitization properties of polyphenol-rich extracts such as honeybee propolis or individual plant polyphenols in cancer have been studied for several years [8]. Caffeic acid, caffeic acid phenethyl ester (CAPE (**1a**, Figure 1)), and ferulic acid, primary phenolic compounds found in honeybee propolis, exhibit anticancer properties in different types of cancer including gliomas [9,10]. Curcumin (diferuloylmethane, **2**, Figure 1), a major ingredient of turmeric (*Curcuma longa* L.), is used in herbal medicine for the treatment of various conditions including inflammation and infection [11]. It has demonstrated potent anticancer activities in diverse models of carcinogenesis notably via targeting cancer-relevant signaling nodes including PI3K and mTOR [12]. Curcumin can also inhibit glioma tumor growth in mouse xenograft models [13]. Thus, such compounds provide interesting starting points in the quest of identifying novel therapeutic agents in brain tumors.

Recent studies conducted in our laboratory have shown CAPE (**1a**) to be significantly more potent than the 5-lipoxygenase (5-LO) clinical inhibitor Zileuton (**3**) for the inhibition of leukotriene (LT) biosynthesis in human polymorphonuclear leukocytes (PMNL, IC_50_ = 0.52 μM) [14]. LTs are a class of eicosanoid inflammatory mediators which have been shown to play an important role in several inflammatory disorders [15]. Moreover, selected malignant cells highly express 5-LO and related LT biosynthesis enzymes, supporting anti-LT therapy as promising anticancer strategy [15,16,17,18,19,20,21]. In response to the need for drugs targeting the LT biosynthesis pathway, studies have been undertaken to develop efficient 5-LO inhibitors [22,23]. Interestingly, a prenylated pyrazole analogue of curcumin (**2**) also selectively inhibited 5-LO and even outperformed curcumin by a factor of approximately 50 [24]. Given that Zileuton (**3**), the only 5-LO inhibitor currently approved for human therapy, requires large and frequent dosing due to an unfavourable pharmacokinetic profile and can contribute to the production of metabolites with hepatotoxic properties [15,25,26,27], it is worthwhile to further investigate alternative options such as CAPE (**1a**).

**Figure 2:**
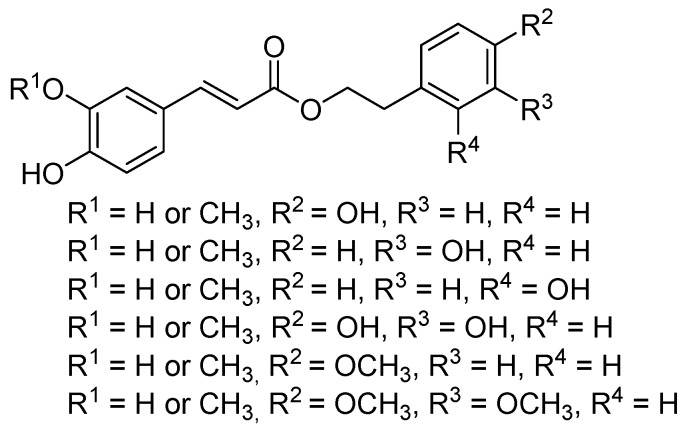
Analogs synthesized in this study.

We recently showed that the ester of caffeic acid CAPE (**1a**) is essential for 5-LO inhibition and that the presence of the two hydroxyls is also important [14]. Building on these findings, the present study evaluates the addition of oxygens moieties (OH or CH_3_O) as substituents on the phenyl group of CAPE (**1a**) and ferulic acid phenethyl ester (FAPE, (**1b**), Figure 1) (Figure 2). The addition of a methoxy substituent, combined with ferulic acid moiety, can be considered as mimic of curcumin (**2**).

All compounds were tested for their ability to inhibit 5-LO. Synthesized compounds were also investigated in two glioma cell lines for their cytotoxic properties and for their sensitizing potential to temozolomide (TMZ, **4**), the primary chemotherapeutic agent utilized in GBM standard of care. Results notably identify a sub-set of compounds with significant 5-LO inhibitory activities and cytotoxic properties towards GBM cells.

## 2. Results and Discussion

### 2.1. Chemistry

**Scheme 1:**
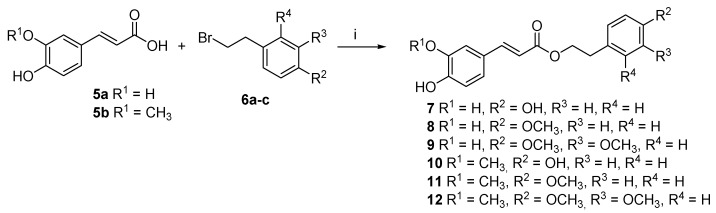
Synthesis of (**7**–**12**). Reagents and conditions: (i), Na_2_CO_3_, BrCH_2_CH_2_Ph(OH), KI, HMPA, r.t., 12 h.

**Scheme 2:**
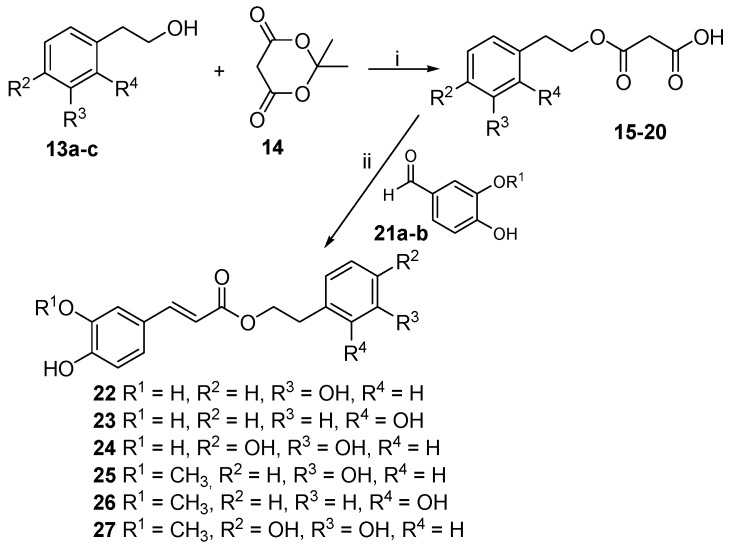
Synthesis of (**22**–**27**). Reagents and conditions: (i) Dioxane, reflux, 5–6 h; (ii) Piperidine, pyridine, r.t., 12 h.

The strategy for synthesis of the alkyl esters is outlined in Scheme 1 and Scheme 2.

Compounds **7**–**12** were prepared by esterification of caffeic acid (**5a**) or ferulic acid (**5b**) with commercially available hydroxy or methoxy phenethyl bromide (**6a**–**c**) in the presence of sodium carbonate (Scheme 1); the overall yield was about 70–80%. This one step synthesis strategy has the advantage of avoiding protection, deprotection, and acyl chloride derivatives synthesis steps.

Attempts to prepare non-commercially available hydroxy or methoxy phenethyl bromide analogs from the corresponding phenethyl alcohols and HBr did not provide the phenethyl bromides in any acceptable yields, only styrene analogs were detected. Appel reaction [28] using triphenylphosphine and tetrabromethane with hydroxy or methoxy phenethyl alcohols which can convert an alcohol to the corresponding alkyl halide under mild conditions did not provide the corresponding bromides.

In a second strategy and as shown in Scheme 2, hydroxy or methoxy phenethyl alcohols (**13a**–**c**) were condensed with Meldrum’s acid (**14**) to provide the malonic acid phenethyl monoesters (**15**–**20**). Knoevenagel condensation of malonic acid phenethyl monoesters (**15**–**20**) with 3,4-dihydroxybenzaldehyde **21a** or 4-hydroxy-3-methoxybenzaldehyde **21b** in pyridine gave compounds **22**–**27** in moderate to good yields.

### 2.2. Biological Evaluation

#### 2.2.1. Inhibition of 5-LO Activity

**Figure 3:**
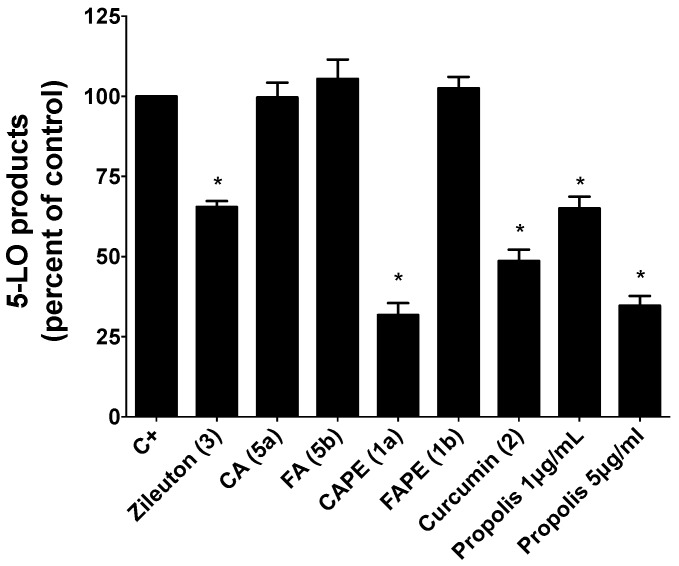
Inhibition of 5-LO product synthesis in stimulated HEK293 cells. Cells were pre-incubated with 1 µM of the indicated compounds or with propolis prior to stimulation as described in the Methods. Values are the means ± SEM of three independent experiments, each performed in duplicate. * different from control (C+) as determined by one way ANOVA with Dunnett’s multiple comparison test (*p* < 0.05).

Natural polyphenolic compounds derived from CAPE (**1a**) have notably been linked to antioxidant and radical scavenging properties [14]. Numerous studies have also highlighted the capacity of these compounds to inhibit 5-LO activity [14,28,29,30,31,32]. Interestingly, strong 5-LO expression has been observed in glioma cells and in primary glioma specimens [20,29]. In addition, inhibiting this enzyme could impact glioma cell proliferation [21]. Compounds were thus investigated for their ability to inhibit leukotriene biosynthesis in HEK293 cells stably transfected with 5-LO and FLAP as a model to investigate 5-LO inhibition [14,30]. As shown in Figure 3, caffeic (CA, **5a**) and ferulic acid (FA, **5b**) had no effect on LT biosynthesis at 1 µM, which mirror results previously reported for caffeic acid in this cell model and in human neutrophils [14,30]. The complete lack of activity for caffeic acid may stem from its relatively hydrophilic nature. As expected, cell treatment with 1 µM of CAPE (**1a**) reduced 5-LO activity by approximately 75% and this was markedly better than Zileuton (**3**), consistent with previous reports [14,30]. The analogue **1b** prepared with ferulic acid had no effect on the biosynthesis of 5-LO products. However curcumin, a natural polyphenol which can be seen as a methylene ketone dimer of ferulic acid, had moderate 5-LO inhibition activity, with approximately 50% inhibition at 1 µM, while the ethanol extract of propolis (a mixture of polyphenols) inhibited 5-LO in a dose-dependent manner with a 35% and 65% reduction of 5-LO activity at 1 and 5 µg/mL, respectively (Figure 3).

**Figure 4:**
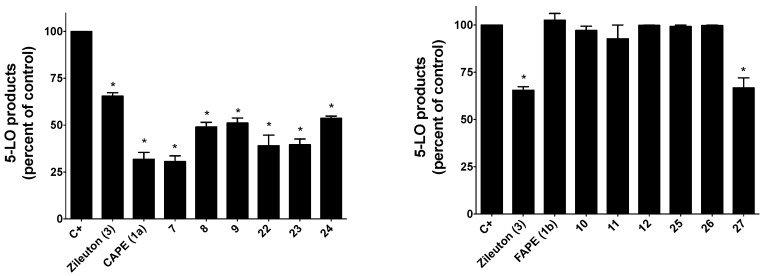
Inhibition of 5-LO product synthesis in stimulated HEK293 cells. Cells were pre-incubated with 1 µM of the indicated compounds prior to stimulation as described in the Methods. Values are the means ± SEM of three independent experiments, each performed in duplicate. * different from control (C+) as determined by one way ANOVA with Dunnett’s multiple comparison test (*p* < 0.05).

The preparation of less polar esters of caffeic acid tends to produce compounds that have moderate to excellent lipoxygenase inhibition [14,31,32]. As shown in Figure 4, esterification of caffeic acid with various phenethyl alcohol derivatives (**7** [33], **8** [33], **9**, **22**, **23** [33] and **24** [34]) produced compounds with notable inhibitory activity at 1 µM, ranging from 50 to 70% inhibition of 5-LO product synthesis. It is noteworthy that inhibition values are fairly insensitive to modifications of the phenethyl portion of these compounds, with only moderate reduction in inhibition even with the bulky 3,4-dimethoxy substitution (**9**) and highly polar 3,4-dihydroxy substitution (**24**). Interestingly, the preparation of analogous esters of ferulic acid (**10**, **11**, **12, 25**, **26** and **27**) essentially abolished inhibitory activity, with one notable exception being **27**, the 3,4-dihydroxyphenethyl ester of ferulic acid (Figure 4).

It has been suggested that phenolic inhibitors of 5-LO derive their inhibitory activity from antioxidant/antiradical pathways by uncoupling the ferric/ferrous redox cycle necessary for 5-LO activity [35]. Accordingly, previous studies have shown ferulic acid to have significantly lower antioxidant/antiradical activity when compared to caffeic acid [36,37,38], a characteristic that would naturally carry over to esterified derivatives of theses acids and hinder their 5-LO inhibitory activity. That **27** retains a certain potency while other ferulic esters do not could stem from the presence of the 3,4-dihydroxyphenethyl ester group, which should permit the formation of orthoquinone type metabolites analogous to caffeic acid metabolites in oxidative settings. Paradoxically, **24**, the 3,4-dihydroxyphenethyl ester of caffeic acid, was the least active of the prepared caffeic esters possibly because the loss of hydrophobicity of the phenethyl ester moiety in comparison to CAPE (**1a**), as this hydrophobic moiety contributes to CAPE’s inhibitory activity as has been suggested previously [14].

#### 2.2.2. Molecular Docking

**Figure 5:**
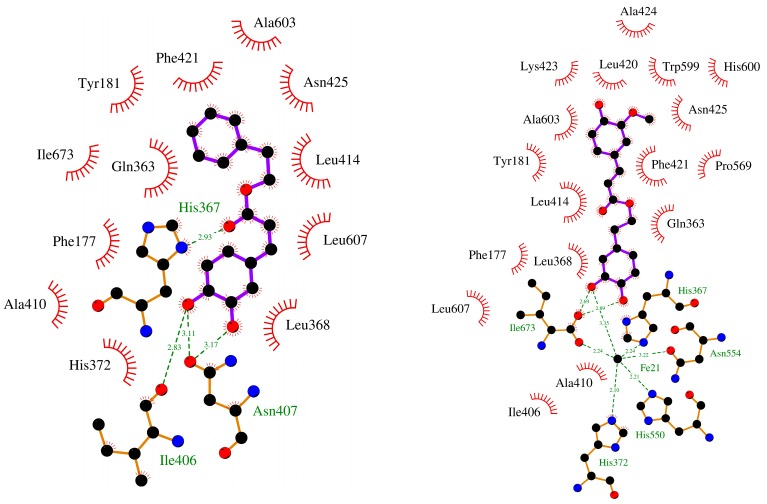
3D images of CAPE (**1a**) (**left**) and **27** (**right**) taken from LigPlot^+^.

Specific compounds were investigated for their ability to interact with 5-LO. Binding energy of the tested compounds revealed substantial stability for **8** (Table 1). Compounds **27**, CAPE (**1a**) and FAPE (**1b**) also displayed, to a lesser extent, strong binding energies. Zileuton (**3**) enantiomers exhibited the lowest binding energies of the investigated compounds (Table 1). Tested molecules adopted three different conformations when interacting with 5-LO, with CAPE (**1a**) (Figure 5) and **8** displaying substantial alignment. Both compounds direct their ‘catechol rings’ in proximity to the iron atom while the rest of the molecule points in the direction of His600. These molecules also participate in similar hydrogen bonds with iron-coordinating residues Asn407 and His367. The interaction of **8** with 5-LO also exhibits two π-π interactions.

The aromatic group of zileuton is aligned with the catechol of CAPE (**1a**) and **8**. Its linear chain points towards the end of the pocket. Both enantiomers display comparable interactions with 5-LO, forming hydrogen bonds with His367 and Gln363 and being involved in a π-π interaction with Phe177.

The alignment observed for the interaction of **7** with 5-LO is comparable to the one observed for CAPE (**1a**) and **8**. However, the interaction is inverted. The catechol group is positioned near the end of the pocket, while the ester double bonded oxygen that points upward for CAPE (**1a**) points in the opposite direction for **7**. Interactions observed with CAPE (**1a**) are also seen with **7** including the hydrogen bonds and a π-π interaction with Phe421.

FAPE (**1b**) and **27** also possess the inverted position observed with **7**, placing the catechol in the vicinity of His600. Compound **27** thus positions its catechol group in a different orientation than the one observed for CAPE (**1a**) (Figure 5). This leads to hydrogen bonds with Ile673, an iron coordinating residue. **27** also forms unique π-π interactions with His367 and Tyr181. FAPE (**1b**), while forming a π-π interaction with His372, does not seem to participate in hydrogen bonding with 5-LO.

#### 2.2.3. Cytotoxic Activity of Compounds

**Figure 6:**
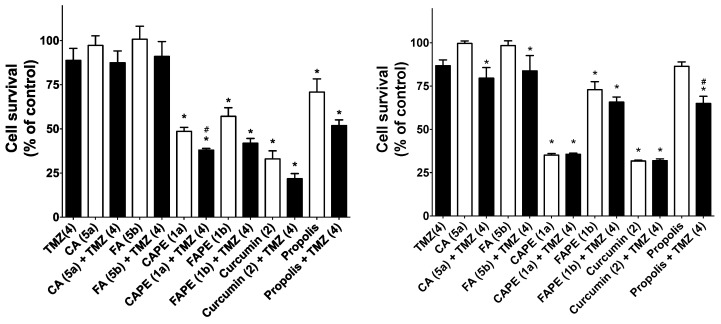
Cell viability of Hs683 (**left panel**) and LN319 (**right panel**) cells following treatment with compounds as described in the Methods. Values are the means ± SEM of three independent experiments, each performed in quadruplicate. * different from control (diluent) as determined by one way ANOVA with Dunnett’s multiple comparison test (*p* < 0.05). **^#^** different from treatment with compound in absence of TMZ as determined by two-sided *t*-test (*p* < 0.05).

**Figure 7:**
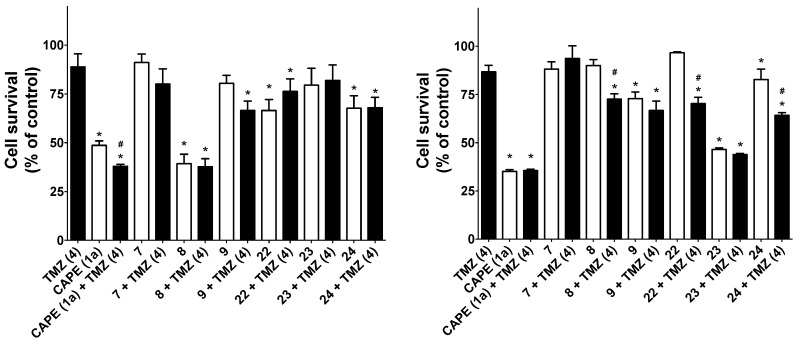
Hs683 (**left panel**) and LN319 (**right panel**) cell viability following treatment with CAPE (**1a**) analogs alone or in combination with TMZ as described in the Methods. Values are the means ± SEM of three independent experiments, each performed in quadruplicate. * different from control (diluent) as determined by one way ANOVA with Dunnett’s multiple comparison test (*p* < 0.05). **^#^** different from treatment with compound in absence of TMZ as determined by two-sided *t*-test (*p* < 0.05).

**Figure 8:**
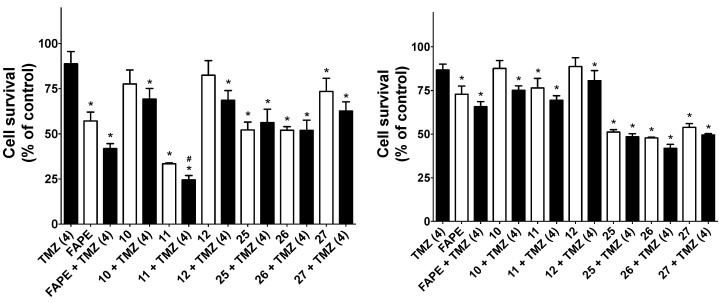
Hs683 (**left panel**) and LN319 (**right panel**) cell viability following treatment with FAPE (**1b**) analogs alone or in combination with TMZ as described in the Methods. Values are the means ± SEM of three independent experiments, each performed in quadruplicate. * different from control (diluent) as determined by one way ANOVA with Dunnett’s multiple comparison test (*p* < 0.05). **^#^** different from treatment with compound in absence of TMZ as determined by two-sided *t*-test (*p* < 0.05).

All the compounds presented in this study were evaluated for their cytotoxic properties in two human glioma cell lines, Hs683 and LN319. As shown in Figure 6, CAPE (**1a**) and FAPE (**1b**) lead to cytotoxicity in Hs683 and LN319 cells, although FAPE (**1b**) was less effective in LN319 cells. These results are in line with earlier studies on CAPE’s effect in gliomas that revealed its ability to negatively impact cell growth and induce apoptosis [39]. FAPE (**1b**) displayed cytotoxic effects in both cell models even though it did not inhibit 5-LO product synthesis suggesting these effects were 5-LO independent. Data is sparse regarding FAPE molecular targets and further investigation of such targets is warranted to elucidate mechanisms underlying the cytotoxic effects of this compound in gliomas. Various studies have also highlighted the ability of curcumin to promote glioma cell apoptosis as well as to reduce cell growth and migration [40,41]. Curcumin (**2**) treatment caused significant cytotoxicity in Hs683 and LN319 leading to cell survival reduction of 68% in both models (Figure 6). Certain synthesized compounds also showed appreciable cytotoxicity in at least one of the cell lines investigated with less than 50% cell survival measured with compounds **8**, **11**, **23** and **26** (Figure 7 and Figure 8). Cytotoxic impact of synthetized compounds were evaluated at different concentrations in both cell lines and calculated LC_50_ values are presented in Table 2.

Every compound used in this study was also evaluated for its capacity to sensitize glioma cells to TMZ (**4**). Treatment with TMZ (**4**) alone yielded only modest impact on viability of both cell lines. Similar resilience to treatments using comparable or greater TMZ concentrations has been observed in Hs683 and LN319 cells by multiple research groups [42,43,44]. While most of the compounds displayed negligible to modest TMZ-sensitizing properties, combinatorial treatment of some compounds with TMZ showed significantly enhanced cytotoxicity compared to treatment with the compounds alone in both glioma models. This was the case for propolis which is in line with previous reports showing that combinatorial treatment of propolis and TMZ (**4**) could inhibit GBM cells growth to a greater extent than when both compounds were used alone [45,46].

Investigating the molecular levers via which this combinatorial effect is exerted should be examined more carefully. In addition, while this work studied the impact of various compounds in one low- and one high-grade glioma cell lines, exploring the effect of select compounds in additional models such as brain-tumor initiating cell lines [47] or normal human astrocytes are envisioned and would build upon the results presented here.

## 3. Conclusions

In summary, we report the design, synthesis, and biological evaluation of CAPE (**1a**) and FAPE (**1b**) analogs having additional oxygens as substituents on the phenethyl group. These analogs can be also considered as new curcumin (**2**) mimics. These compounds were tested for their capabilities to inhibit 5-LO activity as well as for their cytotoxic effects in two glioma cell lines and were compared with drugs approved for 5-LO inhibition and for GBM treatment. CAPE (**1a**), and its analogs **7**, **8**, **9**, **22**, **23** and **24** were capable of substantial 5-LO activity inhibition of at least 50%. FAPE (**1b**) and its analogs **10**, **11**, **12**, **25**, **26**, showed little or no inhibition of 5-LO confirming the importance of the catechol moiety for 5-LO inhibition. The FAPE analogue **27** showed some 5-LO inhibition, suggesting that the presence of vicinal hydroxyl groups on the phenethyl ester moiety can compensate for their absence on the ferulic moiety.

The 5-LO inhibitory activity of the compounds was not predictive of their measured cytotoxic effects as some but not all analogs of both CAPE (**1a**) and FAPE (**1b**) showed cytotoxicity. Interestingly, any structural features associated with cytotoxicity appeared to be related to the substitution on the phenethyl ester moieties. For example, hydroxy substitutions of both CAPE and FAPE analogs at R2 (**7**, **10**) showed little cytotoxicity whereas methoxy substitutions at R2 (**8**, **11**) showed the best cytotoxicity in Hs683 cells. Combining synthesized compounds with TMZ (**4**) in the current study led to some sensitization to the alkylating agent, similar to what is observed with propolis than contains a complex natural mixture of polyphenolic compounds.

Overall, these results provide additional information regarding the anticancer properties associated with CAPE (**1a**) and its analogs. Most importantly, this work highlights novel compounds with significant cytotoxic properties against glioma cell models and further exemplifies the likely importance of exploring the impact of polyphenolic compounds on GBM models.

## 4. Experimental Section

### 4.1. Chemistry

#### 4.1.1. General

All chemicals used were purchased from Aldrich or Alfa Aeser. Propolis was collected in southeast New-Brunswick (Canada) and extraction with ethanol was performed as already described [43]. Purification of compounds was carried out by silica gel circular chromatography (chromatotron^®^, model 7924, Harrison Research, San Bruno, CA, USA) or by flash chromatography (CombiFlash^®^, Separation System SG 100C, ISCO, Lincoln, NE, USA). TLC was run on silica gel coated aluminium sheets (SiliaPlate TLC, Silicycle^®^, Québec, QC, Canada) with detection by UV light (254 nm, UVS-11, Mineralight^®^ shortwave UV lamp). Melting points were obtained using a MELTEMP^®^ (model 1001D) melting point apparatus. FTIR spectra were recorded on a Nicolet^®^ Impact 400 spectrometer. NMR spectra were recorded on a Bruker^®^ Avance III 400 MHz spectrometer. High-resolution mass measurements were performed on a Bruker^®^ Doltonics’ micrOTOF instrument in positive or negative electrospray mode.

#### 4.1.2. Phenolic Acids Esterification: General Procedure (I)

To a solution of caffeic acid (**5a**) or ferulic acid (**5b**) (200–500 mg) in 5 mL of HMPA was added Na_2_CO_3_ (1.2 eq.) and the mixture was stirred for 30 min in an ice bath. Corresponding arylbromide, dissolved in 1 mL of HMPA, was then added drop by drop over 20 min followed by few mg of KI. After 12 h of stirring at room temperature, the reaction mixture was poured in 50 mL of ice water, stirred for 30 min and then extracted with ethyl acetate (3 × 40 mL). The combined organic phases were washed with water (3 × 25 mL), with brine (3 × 25 mL), and dried over MgSO_4_, filtered, and concentrated followed by flash chromatography (0–70% AcOEt/hexanes) purification to give the desired product.

#### 4.1.3. Phenolic Acids Esterification: General Procedure (II)

To a solution of hydroxyphenethyl alcohol and Meldrum’s acid (1.2 eq.), dioxane was refluxed for 5–6 h. After removal of the dioxane, malonic acid mono- or dihydroxyphenethyl monoesters were obtained as yellow, viscous oils which were used in the next step without purification. Piperidine (1 mL) was added to a mixture of malonic acid mono- or dihydroxyphenethyl monoester and 3,4-dihydroxybenzaldehyde (1.5 eq.) in pyridine (20 mL). The mixture was stirred at room temperature for 12 h. The reaction mixture was concentrated under vacuum to produce a residue, which was dissolved in EtOAc (200 mL) and then washed with water (3 × 25 mL), with brine (3 × 25 mL), and dried over MgSO_4_, filtered, and concentrated, followed by flash chromatography (10–70% AcOEt/hexanes) purification to give the desired product.

#### 4.1.4. Caffeic Acid 4-Hydroxyphenethyl Ester (**7**)

Following general procedure I with **5a** (250 mg, 1.38 mmol), **6a** (316 mg, 1.56 mmol), Na_2_CO_3_ (180 mg, 1.69 mmol), and HMPA (5 mL). A white solid was obtained after purification by flash chromatography (EtOAc-hexane 0–40%). Yield = 220 mg (53%), m.p. 179–182 °C; IR (ν (cm^−1^)): 3300–3000 (O–H), 1677 (C=O), ^1^H NMR (400 MHz, DMSO-*d*_6_, 25 °C) δ (ppm): 9.59 (1H, s, OH), 9.22 (2H, s, OH), 7.46 (1H, d, *J* = 15.8 Hz, CH), 7.10–7.03 (3H, m, H_arom_), 7.00 (1H, d, *J* = 8.2 Hz, H_arom_), 6.76 (1H, d, *J* = 8.1 Hz, H_arom_), 6.70 (2H, d, *J* = 8.2 Hz, H_arom_), 6.24 (1H, d, *J* = 15.9 Hz, CH), 4.24 (2H, t, *J* = 7.0 Hz, CH_2_), 2.83 (2H, t, *J* = 6.9 Hz, CH_2_). ^13^C NMR (101 MHz, DMSO-*d*_6_, 25 °C) δ (ppm): 166.94, 156.31, 148.87, 146.03, 145.60, 130.24, 128.45, 125.93, 121.83, 116.20, 115.61, 115.29, 114.37, 65.14, 34.16. HRMS *m*/*z* C_17_H_16_O_5_-(H^−^): calcd. for: 299.0925; found: 299.0933.

#### 4.1.5. Ferulic Acid 4-Methoxyphenethyl Ester (**8**)

Following general procedure I with **5b** (500 mg, 2.57 mmol), **6b** (726 mg, 3.37 mmol), Na_2_CO_3_ (410 mg, 3.86 mmol), and HMPA (5 mL). A white solid was obtained after purification by flash chromatography (EtOAc-hexane 0–50%). Yield = 650 mg (77%); m.p. 118–120 °C; IR (ν (cm^−1^)): 3350–3000 (O–H), 1682 (C=O),^1^H NMR (400 MHz, CDCl_3_, 25 °C) δ (ppm): 7.63 (1H, d, *J* = 15.9 Hz, CH), 7.20 (2H, t, *J* = 5.7 Hz, H_arom_), 7.09 (1H, dd, *J* = 1.8 Hz, 8.2 Hz, H_arom_), 7.05 (1H, d, *J* = 1.8 Hz, H_arom_), 6.94 (1H, d, *J* = 8.2 Hz, H_arom_), 6.91–6.86 (2H, m, H_arom_), 6.30 (1H, d, *J* = 15.9 Hz, CH), 5.90 (1H, s, OH), 4.40 (2H, t, *J* = 7.1 Hz, CH_2_), 3.94 (3H, s, CH_3_), 3.82 (3H, s, CH_3_), 2.98 (2H, t, *J* = 7.1 Hz, CH_2_). ^13^C NMR (101 MHz, CDCl_3_, 25 °C) δ (ppm): 167.20, 158.32, 147.97, 146.77, 144.90, 129.96, 129.89, 127.01, 123.11, 115.47, 114.71, 113.95, 109.32, 65.13, 55.96, 55.27, 34.37. HRMS *m*/*z* C_19_H_20_O_5_-(H^−^): calcd. for: 327.1238; found: 327.1238.

#### 4.1.6. Caffeic Acid 3,4-Dimethoxyphenethyl Ester (**9**)

Following general procedure I with **5a** (250 mg, 1.38 mmol), **6c** (385 mg, 1.57 mmol), Na_2_CO_3_ (180 mg, 1.7 mmol), and HMPA (5 mL). A white solid was obtained after purification by flash chromatography (EtOAc-hexane 0–60%). Yield = 300 mg (63%), m.p. 138–140 °C; IR (ν (cm^−1^)): 3350–3000 (O–H), 1675 (C=O), ^1^H NMR (400 MHz, DMSO-*d*_6_, 25 °C) δ (ppm): 9.57 (1H, s br, OH), 9.17 (1H, s br, OH) 7.47 (1H, d, *J* = 15.9 Hz, CH), 7.04–7.00 (2H, m, H_arom_), 6.89–6.87 (2H, d, *J* = 9.0 Hz, H_arom_) 6.80–6.75 (2H, t, *J* = 8.2 Hz, H_arom_) 6.25 (1H, d, *J* = 15.9 Hz, CH), 4.31–4.28 (2H, t, *J* = 6.9 Hz, CH_2_), 3.73 (3H, s, CH_3_), 3.72 (3H, s, CH_3_), 2.90–2.86 (2H, t, *J* = 6.9 Hz, CH_2_). ^13^C NMR (101 MHz, DMSO-*d*_6_, 25 °C) δ (ppm): 166.93, 149.08, 148.89, 147.87, 146.04, 145.61, 130.90, 125.92, 121.82, 121.20, 116.20, 115.29, 114.37, 113.25, 112.33, 65.00, 55.95, 55.86, 34.55. HRMS *m*/*z* C_19_H_20_O_6_-(H^−^): calcd. for: 343.1187; found: 343.118.

#### 4.1.7. Ferulic Acid 4-Hydroxyphenethyl Ester (**10**)

Following general procedure I with **5b** (250 mg, 1.28 mmol), **6a** (293 mg, 1.45 mmol), Na_2_CO_3_ (163 mg, 1.53 mmol), and HMPA (5 mL). An off white solid was obtained after purification by flash chromatography (EtOAc-hexane 0–40%). Yield = 220 mg (54%), m.p. 80–83 °C; IR (ν (cm^−1^)): 3300–3000 (O–H), 1672 (C=O), ^1^H NMR (400 MHz, CDCl_3_, 25 °C) δ (ppm): 7.63 (1H, d, *J* = 15.9 Hz, CH), 7.14 (2H, d, *J* = 8.5 Hz, H_arom_), 7.09 (1H, dd, *J* = 1.8Hz, 8.2 Hz, H_arom_), 7.04 (1H, d, *J* = 1.8 Hz, H_arom_), 6.94 (1H, d, *J* = 8.2 Hz, H_arom_), 6.84–6.80 (2H, m, H_arom_), 6.30 (1H, d, *J* = 15.8 Hz, CH), 5.86 (1H, s OH), 5.19 (1H, s, OH), 4.40 (2H, t, *J* = 7.1 Hz, CH_2_), 3.94 (3H, s, CH_3_), 2.97 (2H, t, *J* = 7.1 Hz, CH_2_). ^13^C NMR (101 MHz, CDCl_3_, 25 °C) δ (ppm): 167.44, 154.36, 148.00, 146.77, 145.12, 130.07, 129.94, 126.96, 123.15, 115.41, 115.34, 114.73, 109.37, 65.22, 55.97, 34.35. HRMS *m*/*z* C_18_H_18_O_5_-(H^−^): calcd. for: 313.1081; found: 313.1076.

#### 4.1.8. Caffeic Acid 4-Methoxyphenethyl Ester (**11**)

Following general procedure I with **5a** (500 mg, 2.7 mmol), **6b** (776 mg, 3.61 mmol), Na_2_CO_3_ (430 mg, 4 mmol), and HMPA (5 mL). A white solid was obtained after purification by flash chromatography (EtOAc-hexane 0–60%). Yield = 600 mg (69%), m.p. 185–186 °C; IR (ν (cm^−1^)): 3400–3000 (O–H), 1698 (C=O), ^1^H NMR (400 MHz, DMSO-*d*_6_, 25 °C) d (ppm): 9.63 (1H, br s, OH), 9.17 (1H, br s, OH), 7.46 (1H, d, *J* = 15.8 Hz, CH), 7.20 (2H, d, *J* = 8.3 Hz, H_arom_), 7.04 (1H, s, H_arom_), 7.00 (1H, d, *J* = 8.2 Hz, H_arom_), 6.87 (2H, d, *J* = 8.3 Hz, H_arom_), 6.76 (1H, d, *J* = 8.1 Hz, H_arom_), 6.24 (1H, d, *J* = 15.9 Hz, CH), 4.27 (2H, t, *J* = 6.8 Hz, CH_2_), 3.72 (3H, s, OCH_3_), 2.88 (2H, t, *J* = 6.8 Hz, CH_2_); ^13^C NMR (101 MHz, DMSO-*d*_6_, 25 °C) d (ppm): 166.95, 158.31, 148.88, 146.02, 145.64, 130.33, 125.90, 121.86, 116.18, 115.27, 114.31, 114.25, 65.03, 55.43, 34.07; HRMS *m*/*z* C_18_H_18_O_5_+(K^+^): calcd. for: 353.0786; found: 353.0772.

#### 4.1.9. Ferulic Acid 3,4-Dimethoxyphenethyl Ester (**12**)

Following general procedure I with **5b** (200 mg, 1.03 mmol), **6c** (286 mg, 1.16 mmol), Na_2_CO_3_ (133 mg, 1.25 mmol), and HMPA (5 mL). A white solid was obtained after purification by flash chromatography (EtOAc-hexane 0–60%). Yield = 250 mg (68%), m.p. 126–129 °C; IR (ν (cm^−1^)): 3300–3000 (O–H), 1696 (C=O), ^1^H NMR (400 MHz, DMSO-*d*_6_, 25 °C) δ (ppm): 9.61 (1H, s, OH), 7.55 (1H, d, *J* = 15.9 Hz, CH), 7.32 (1H, s, H_arom_), 7.11 (1H, d, *J* = 8.1 Hz, H_arom_), 6.88 (2H, d, *J* = 12.2 Hz, H_arom_), 6.80 (2H, d, *J* = 7.7 Hz, H_arom_), 6.46 (1H, d, *J* = 15.9 Hz, CH), 4.32 (2H, t, *J* = 6.7 Hz, CH_2_), 3.82 (3H, s, CH_3_), 3.74 (3H, s, CH_3_), 3.72 (3H, s, CH_3_), 2.89 (2H, t, *J* = 6.6 Hz, CH_2_); ^13^C NMR (101 MHz, DMSO-*d*_6_, 25 °C) δ (ppm): 167.04, 149.83, 149.09, 148.41, 147.88, 145.56, 130.87, 126.01, 123.65, 121.17, 115.96, 114.87, 113.23, 112.33, 111.64, 64.96, 56.16, 55.95, 55.86, 34.54. HRMS *m*/*z* C_20_H_22_O_6_-(H^−^): calcd. for: 357.1344; found: 357.1341.

#### 4.1.10. Caffeic Acid 3-Hydroxyphenethyl Ester (**22**)

Following general procedure II with **13a** (500 mg, 3.61 mmol), **14** (694 mg, 4.81 mmol), dioxane (10 mL), **21a** (440 mg, 3.18 mmol), piperidine (1 mL), and pyridine (20 mL). An off white solid was obtained after purification by flash chromatography (EtOAc-hexane 0–60%). Yield = 230 mg (36%), m.p. 138–142 °C; IR (ν (cm^−1^)): 3300–3000 (O–H), 1688 (C=O), ^1^H NMR (400 MHz, MeOH-*d*_4_, 25 °C) δ (ppm): 7.54 (1H, d, *J* = 15.9 Hz, CH), 7.13 (1H, t, *J* = 7.8 Hz, H_arom_) 7.05 (1H, d, *J* = 2.0 Hz, H_arom_), 6.95 (1H, dd, *J* = 2.0 Hz, 8.2 Hz, H_arom_), 6.80 (1H, d, *J* = 8.2 Hz, H_arom_), 6.70 (3H, m, H_arom_), 6.25 (1H, d, *J* = 15.9 Hz, CH), 4.36 (2H, t, *J* = 7.0 Hz, CH_2_), 2.93 (2H, t, *J* = 7.0 Hz, CH_2_). ^13^C NMR (101 MHz, MeOH-*d*_4_, 25 °C) δ (ppm): 167.87, 157.12, 148.17, 145.58, 145.39, 139.49, 129.11, 126.32, 121.59, 119.79, 115.42, 115.11, 113.76, 113.69, 113.07, 64.74, 34.75. HRMS *m*/*z* C_17_H_16_O_5_-(H^−^): calcd. for: 299.0925; found: 299.0919.

#### 4.1.11. Caffeic Acid 2-Hydroxyphenethyl Ester (**23**)

Following general procedure II with **13b** (500 mg, 3.62 mmol), **14** (690 mg, 4.78 mmol), dioxane (10 mL), **21a** (440 mg, 3.18 mmol), piperidine (1 mL), and pyridine (20 mL). A greyish solid was obtained after purification by flash chromatography (EtOAc-hexane 0–60%). Yield = 220 mg (34%), m.p. 137–142 °C; IR (ν (cm^−1^)): 3400–3000 (O–H), 1677 (C=O), ^1^H NMR (400 MHz, MeOH-*d*_4_, 25 °C) δ (ppm): 7.53 (1H, d, *J* = 15.9 Hz, CH), 7.13 (1H, d, *J* = 7.3 Hz, H_arom_), 7.09–7.03 (2H, m, H_arom_), 6.95 (1H, d, *J* = 7.8 Hz, H_arom_), 6.78 (3H, m, H _arom_), 6.24 (1H, d, *J* = 15.9 Hz, CH), 4.37 (2H, t, *J* = 7.0 Hz, CH_2_), 3.00 (2H, t, *J* = 7.0 Hz, CH_2_). ^13^C NMR (101 MHz, MeOH-*d*_4_, 25 °C) δ (ppm): 168.02, 155.32, 148.24, 145.42, 130.47, 127.38, 127.31, 126.31, 123.96, 121.53, 119.10, 115.10, 114.53, 113.80, 113.69, 63.63, 29.59. HRMS *m*/*z* C_17_H_16_O_5_-(H^−^): calcd. for: 299.0925; found: 299.0921.

#### 4.1.12. Caffeic Acid 3,4-Dihydroxyphenethyl Ester (**24**)

Following general procedure II with **13c** (370 mg, 2.4 mmol), **14** (460 mg, 3.2 mmol), dioxane (10 mL), **21a** (510 mg, 3.69 mmol), piperidine (1 mL), and pyridine (20 mL). A brown solid was obtained after purification by flash chromatography (EtOAc-hexane 0–70%). Yield = 200 mg (50%), m.p. 76–79 °C; IR (ν (cm^−1^)): 3300–3000 (O–H), 1676 (C=O), ^1^H NMR (400 MHz, MeOH-*d*_4_, 25 °C) δ (ppm): 7.54 (1H, d, *J* = 15.8 Hz, CH), 7.05 (1H, d, *J* = 1.5 Hz, H_arom_), 6.95 (1H, d, *J* = 8.1 Hz, H_arom_), 6.79 (1H, d, *J* = 8.1 Hz, H_arom_), 6.72 (2H, dd, *J* = 3.1 Hz, 4.8 Hz, H_arom_), 6.59 (1H, d, *J* = 8.0 Hz, H_arom_), 6.25 (1H, d, *J* = 15.9 Hz, CH), 4.31 (2H, t, *J* = 7.0 Hz, CH_2_), 2.85 (2H, t, *J* = 6.9 Hz, CH_2_). ^13^C NMR (101 MHz, MeOH-*d*_4_, 25 °C) δ (ppm): 167.87, 148.16, 145.50, 145.40, 144,86, 143.52, 129.44, 126.33, 121.54, 119.83, 115.66, 115.09, 115.00, 113.76, 65.11, 34.20. HRMS *m*/*z* C_17_H_16_O_6_+(Br^−^): calcd. for: 397.0118; found: 397.0122.

#### 4.1.13. Ferulic Acid 3-Hydroxyphenethyl Ester (**25**)

Following general procedure II with **13a** (500 mg, 3.61 mmol), **14** (695 mg, 4.81 mmol), dioxane (10 mL), **21b** (520 mg, 3.42 mmol), piperidine (1 mL), and pyridine (20 mL). A yellow solid was obtained after purification by flash chromatography (EtOAc-hexane 0–50%). Yield = 303 mg (42%), m.p. 138–141 °C; IR (ν (cm^−1^)): 3300–3000 (O–H), 1665 (C=O), ^1^H NMR (400 MHz, CDCl_3_, 25 °C) δ (ppm): 7.60 (1H, d, *J* = 15.9 Hz, CH), 7.21 (1H, t, *J* = 7.8 Hz, H_arom_), 7.15 (1H, d, *J* = 2.0 Hz, H_arom_), 7.05 (1H, dd, *J* = 3.8 Hz, 8.0 Hz, H_arom_), 6.86 (2H, dd, *J* = 3.8 Hz, 8.0 Hz, H_arom_), 6.74 (2H, dd, *J* = 5.5 Hz, 13.6 Hz, H_arom_), 6.30 (1H, d, *J* = 15.9 Hz, CH), 4.42 (2H, t, *J* = 7.1 Hz, CH_2_), 3.95 (3H, s, CH_3_), 2.99 (2H, t, *J* = 7.0 Hz, CH_2_). ^13^C NMR (101 MHz, CDCl_3_, 25 °C) δ (ppm): 167.18, 155.65, 148.51, 145.85, 144.73, 139.86, 129.72, 128.07, 121.84, 121.42, 116.09, 115.87, 113.51, 113.03, 110.51, 64.72, 56.01, 35.08. HRMS *m*/*z* C_18_H_18_O_5_-(H^−^): calcd. for: 313.1081; found: 313.1073.

#### 4.1.14. Ferulic Acid 2-Hydroxyphenethyl Ester (**26**)

Following general procedure II with **13b** (500 mg, 3.62 mmol), **14** (690 mg, 4.8 mmol), dioxane (X mL), **21b** (440 mg, 2.89 mmol), piperidine (1 mL), and pyridine (20 mL). A light brown solid was obtained after purification by flash chromatography (EtOAc-hexane 0–50%). Yield = 395 mg (47%), m.p. 109–112 °C; IR (ν (cm^−1^)): 3300–3100 (O–H), 1667 (C=O), ^1^H NMR (400 MHz, CDCl_3_, 25 °C) δ (ppm): 7.65 (1H, d, *J* = 15.9 Hz, CH), 7.21–7.13 (3H, m, H_arom_), 7.05 (1H, d, *J* = 8.3 Hz, H_arom_), 6.92–6.84 (3H, m, H_arom_), 6.31 (1H, d, *J* = 15.9 Hz, CH), 4.42 (2H, t, *J* = 6.9 Hz, CH2), 3.95 (3H, s, CH_3_), 3.06 (2H, t, *J* = 7.1 Hz, CH_2_); ^13^C NMR (101 MHz, CDCl_3_, 25 °C) δ (ppm): 167.91, 154.63, 148.73, 145.88, 145.44, 130.91, 128.33, 127.89, 123.61, 122.04, 120.59, 115.99, 115.59, 113.13, 110.56, 64.45, 56.02, 30.33. HRMS *m*/*z* C_18_H_18_O_5_-(H^−^): calcd. for: 313.1081; found: 313.1067.

#### 4.1.15. Ferulic Acid 3,4-Dihydroxyphenethyl Ester (**27**)

Following general procedure II with **13c** (370 mg, 2.4 mmol), **14** (460 mg, 3.2 mmol), dioxane (10 mL), **21b** (570 mg, 3.75 mmol), piperidine (1 mL), and pyridine (20 mL). A light brown solid was obtained after purification by flash chromatography (EtOAc-hexane 0–70%). Yield = 310 mg (37%), m.p. 132–136 °C; IR (ν (cm^−1^)): 3400–3100 (O–H), 1685 (C=O), ^1^H NMR (400 MHz, MeOH-*d*_4_, 25 °C) δ (ppm): 7.56 (1H, d, *J* = 15.9 Hz, CH), 7.09–7.04 (2H, m, H_arom_), 6.95 (1H, d, *J* = 8.2 Hz, H_arom_), 6.72 (2H, dd, *J* = 3.0 Hz, 4.9 Hz, H_arom_), 6.60 (1H, dd, *J* = 1.9 Hz, 8.0 Hz, H_arom_), 6.30 (1H, d, *J* = 15.9 Hz, CH), 4.32 (2H, t, *J* = 7.1 Hz, CH_2_), 3.90 (3H, s, CH_3_), 2.83 (2H, t, *J* = 7.0 Hz, CH_2_); ^13^C NMR (101 MHz, MeOH-*d*_4_, 25 °C) δ (ppm): 167.72, 150.10, 146.59, 145.11, 144.86, 143.53, 129.44, 127.47, 121.39, 119.85, 115.67, 115.03, 114.78, 113.35, 111.11, 65.18, 54.98, 34.17. HRMS *m*/*z* C_18_H_18_O_6_-(H^−^): calcd. for: 329.1031; found: 329.1023.

### 4.2. 5-Lipoxygenase Activity Assay

HEK293 cells stably co-transfected with a pcDNA3.1 vector expressing 5-LO and a pBUDCE4.1 vector expressing 5-LO activating protein (FLAP) were utilized to screen compounds for 5-LO inhibition [14,32] as previously described. Briefly, for cell stimulation of 5-LO products, transfected HEK293 cells were collected following trypsinization, washed and the cell pellet was resuspended in Hank’s balanced salt solution (HBSS) (Lonza, Walkerville, MD, USA) containing 1.6 mM CaCl_2_ at a concentration of 5 × 10^5^ cells mL^−1^. Cells were pre-incubated with each test compound at 1 µM for 5 min at 37 °C. Cells were then stimulated for 15 min at 37 °C with the addition of 10 µM calcium ionophore A23187 (Sigma–Aldrich, Oakville, ON, Canada) and 10 µM arachidonic acid (Cayman Chemical, Ann Arbor, MI, USA). Stimulations were stopped by the addition of 0.5 volume of cold MeOH:CH_3_CN (1:1) containing 50 ng of PGB_2_ as internal standard and samples were stored at −20 °C until processing on octadecyl (C18) columns and analysis by RP-HPLC as described previously [48]. Data are expressed as means ± SEM of three independent experiments, each performed in duplicate.

### 4.3. Molecular Docking

Molecular docking was performed with AutoDock 4.0, Autogrid [49] and AutoDock Tools [50]. Standard AutoDock protocol was followed unless otherwise noted. Ligands were drawn and processed with AutoDock Tools for charge and rotatable bonds assignment. 5-LO crystal structure (PDB ID: 3O8Y [51]), which is a “stable-5-LOX”, was chosen for docking. To enable crystallization, several mutations are present in the non-catalytic domain and a small three residue sequence in the catalytic domain is replaced from KKK to ENL. The mutations maybe affect the structure, but “stable-5-LOX” catalytic activity was not affected [51].

Protein was prepared with AutoDock Tools. Water molecules were removed, polar hydrogens added, and charges assigned. The grid box used a default spacing of 0.375 with a bounding box of (60, 66, 60) and a grid center of (−2.24, 25.69, −0.94), in (X, Y, Z) coordinates. For docking settings, 100 runs were completed per ligand and defaults were kept with the following exceptions: ga_pop_size 5000, ga_num_evals 100,000,000, ga_num_generations 500,000, sw_max_its 5000. For analysis, AutoDock Tools, Maestro [52] and LigPlot^+^ [53] were used. Results were clustered with a maximum of 2.00 Å RMSD and the largest cluster with the lowest binding energy was chosen.

### 4.4. Cytotoxic Effects of Compounds in Hs683 and LN319 Glioma Cells

Human glioma cells Hs683 and LN319 were cultured using Dulbecco’s Modified Eagle Medium (DMEM) supplemented with 10% foetal bovine serum (FBS) and antibiotics (Thermo Fisher Scientific) and have been described elsewhere [54]. Cytotoxic properties of compounds were assessed via a crystal violet assay as before [55]. Briefly, 10,000 cells were seeded in quadruplicates in 96-well plates in 200 μL of cell medium containing compounds at a concentration of 10 μM, 30 μg/mL of propolis or 100 µM TMZ. Compounds were replaced with dimethyl sulfoxide (DMSO) in control wells. Cells were subsequently placed for 3 days at 37 °C and 5% CO_2_ in an incubator. Following incubation, cell medium was removed and cells were rinsed with 200 μL phosphate-buffered saline (PBS). Cells were treated with 100 μL of cold methanol for 10 min and washed with 200 μL of PBS. Crystal violet staining of cells was subsequently performed for 20 min. Crystal violet was removed from the wells and the excess was rinse out with water and the plate was put in the incubator for 20 min to remove the excess of water. Stained cells were re-suspended in 100 μL of 1% sodium dodecyl sulfate (SDS). Absorbance was read at 595 nm using a Varioscan instrument (Scanlab Inc., Munich, Germany). Viable cells were calculated as a percentage of absorbance with respect to control cells. Mean values ± SEM are reported. The cytotoxic impact of compounds at various concentrations were evaluated using the same experimental approach. LC_50_ values were obtained from a sigmoidal concentration–response curve-fitting model with a variable slope using GraphPad Prism 6 software (GraphPad, San Diego, CA, USA).

The ability of compounds to sensitize glioma cells to TMZ was also investigated using this crystal violet assay-based approach. A total of 10,000 cells were seeded in quadruplicates in 96-well plates in 200 μL of cell medium containing 10 µM of compounds, 30 μg/mL of propolis, and 100 µM TMZ. DMSO replaced compounds and TMZ in control wells. Crystal violet staining and quantification was performed as above. Cells deemed viable were calculated as a percentage of absorbance with respect to control cells. Results depict mean values ± SEM.

## Figures and Tables

**Table 1:** Binding energy, bonds, and interactions for individual ligands.

Molecule	Binding Energy (kcal/mol)	Hydrogen Bond	π-π Interactions
CAPE (**1a**)	−8.19	His367, Asn407 × 2	Phe421
FAPE (**1b**)	−8.13	-	His372
**7**	−7.84	Asn407, Asn425	Phe177, Phe421
**8**	−8.59	His367, Asn407 × 2	Phe177, Phe421
**27**	−8.30	Ile673 × 2	Tyr181, His367
Zileuton (**3**, (*R*))	−7.34	Gln363, His367	Phe177
Zileuton (**3**, (*S*))	−7.02	Gln363, His367	Phe1 77

**Table 2:** LC_50_ of molecules obtained following cytotoxic assays on Hs683 and LN319 cell lines. Data shown are means ± SEM.

Molecule	Hs683 (μM)	LN319 (μM)
CAPE (**1a**)	10.9 ± 1.2	4.6 ± 0.7
FAPE (**1b**)	17.4 ± 2.0	20.2 ± 1.5
Curcumin (**2**)	8.0 ± 2.6	2.7 ± 0.1
CA (**5a**)	>400	>400
FA (**5b**)	>400	>400
**7**	35.1 ± 2.2	17.9 ± 1.5
**8**	1.4 ± 1.3	24.9 ± 1.8
**9**	18.7 ± 1.4	43.5 ± 6.3
**10**	22.8 ± 1.7	38.0 ± 7.0
**11**	3.3 ± 1.2	24.3 ± 8.9
**12**	130.1 ± 2.8	>400
**22**	22.7 ± 1.8	52.6 ± 13.5
**23**	18.4 ± 1.1	8.5 ± 1.6
**24**	54.1 ± 2.2	37.1 ± 7.6
**25**	13.1 ± 1.8	11.4 ± 3.2
**26**	8.7 ± 1.1	5.3 ± 2.1
**27**	22.9 ± 1.2	13.6 ± 2.2
Propolis	87.5 μg/mL ± 4.9 μg/mL	87.6 μg/mL ± 5.8 μg/mL
